# Supplementary material for: Characterization of the fungal community in the canopy air of the invasive plant Ageratina adenophora and its potential to cause plant diseases
Source: PLoS One. 2020 Mar 26;15(3):e0230822. doi: 10.1371/journal.pone.0230822 (PMC7098561; doi:10.1371/journal.pone.0230822)
Supplement: S1 Fig — LC, low part of canopy air of A. adenphora in the invaded region; HC, high part of canopy air of A. adenphora in the invaded region; YL, samples from Yunlong county (invaded region); KM, samples from Kunming city (invaded region). (DOCX) [file pone.0230822.s001.docx]

**
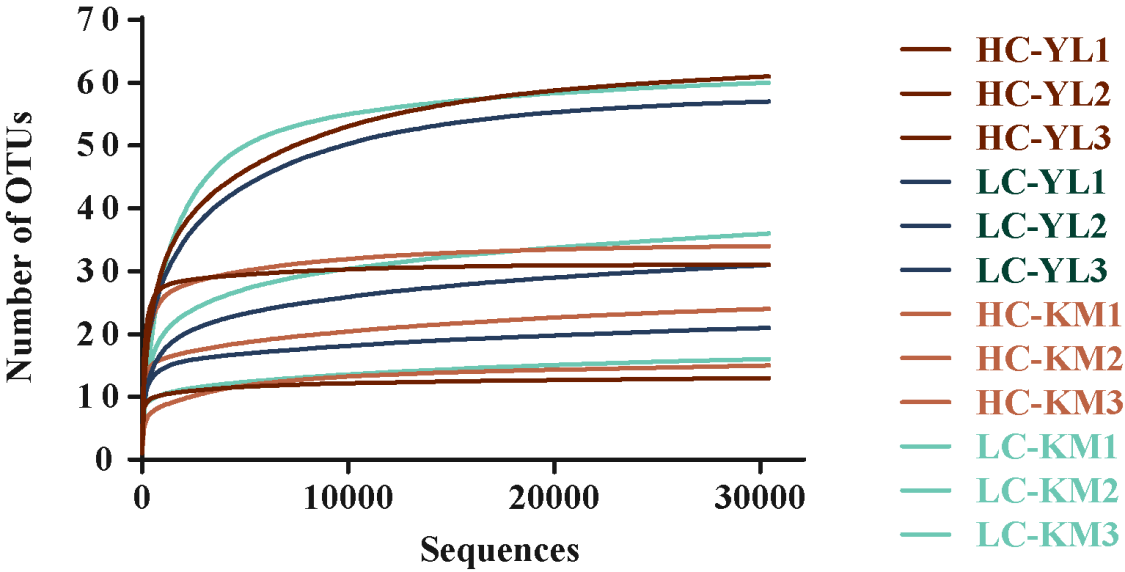
**

**S1 Fig. Rarefaction curves.** LC, low part of canopy air of *A. adenphora* in the invaded region; HC, high part of canopy air of *A. adenphora* in the invaded region; YL, samples from Yunlong county (invaded region); KM, samples from Kunming city (invaded region).
