# Supplementary material for: Characterization of the fungal community in the canopy air of the invasive plant Ageratina adenophora and its potential to cause plant diseases
Source: PLoS One. 2020 Mar 26;15(3):e0230822. doi: 10.1371/journal.pone.0230822 (PMC7098561; doi:10.1371/journal.pone.0230822)
Supplement: S1 Table — (DOCX) [file pone.0230822.s002.docx]

**S1 Table. Sampling sites information**

| Sampling site | Latitude(N) | Longitude(E) | Elevation(m) |
| --- | --- | --- | --- |
| KM1 | 24°55′34″ | 102°38′30″ | 1890 |
| KM2 | 24°54′44″ | 102°38′55″ | 1890 |
| KM3 | 24°58′22″ | 102°27′49″ | 1980 |
| YL1 | 25°58′4″ | 99°19′37″ | 1730 |
| YL2 | 25°53′1″ | 99°25′36″ | 2110 |
| YL3 | 25°42′16″ | 99°37′56″ | 1950 |
